# Phytoplankton gross primary production increases along cascading impoundments in a temperate, low-discharge river: Insights from high frequency water quality monitoring

**DOI:** 10.1038/s41598-019-43008-w

**Published:** 2019-04-30

**Authors:** Fabian Engel, Katrin Attermeyer, Ana I. Ayala, Helmut Fischer, Volker Kirchesch, Don C. Pierson, Gesa A. Weyhenmeyer

**Affiliations:** 1 Department of Ecology and Genetics/Limnology, Uppsala University, Norbyvägen 18D, 752 36 Uppsala, Sweden; 2 Present Address: WasserCluster Lunz Biologische Station GmbH, Dr. Carl Kupelwieser Promenade 5, 3293 Lunz am See, Austria; 3 Department of Microbial Ecology, German Federal Institute of Hydrology (BfG), Am Mainzer Tor 1, 56068 Koblenz, Germany

**Keywords:** Carbon cycle, Environmental sciences, Limnology

## Abstract

Damming alters carbon processing along river continua. Estimating carbon transport along rivers intersected by multiple dams requires an understanding of the effects of cascading impoundments on the riverine metabolism. We analyzed patterns of riverine metabolism and phytoplankton biomass (chlorophyll *a*; Chl*a*) along a 74.4-km river reach intersected by six low-head navigation dams. Calculating gross primary production (GPP) from continuous measurements of dissolved oxygen concentration, we found a maximum increase in the mean GPP by a factor of 3.5 (absolute difference of 0.45 g C m^−3^ d^−1^) along the first 26.5 km of the study reach, while Chl*a* increased over the entire reach by a factor of 2.9 (8.7 µg l^−1^). In the intermittently stratified section of the deepest impoundment the mean GPP between the 1 and 4 m water layer differed by a factor of 1.4 (0.31 g C m^−3^ d^−1^). Due to the strong increase in GPP, the river featured a wide range of conditions characteristic of low- to medium-production rivers. We suggest that cascading impoundments have the potential to stimulate riverine GPP, and conclude that phytoplankton CO_2_ uptake is an important carbon flux in the river Saar, where a considerable amount of organic matter is of autochthonous origin.

## Introduction

River systems play an important role in the carbon transport between terrestrial ecosystems, the atmosphere and the ocean, and thus in the global carbon cycle^1–3^. Large amounts of carbon are transformed in rivers along the aquatic continuum from land to sea^4^, and about 0.65 Pg C are annually emitted from rivers to the atmosphere as CO_2_^5^. Dam construction affects more than half of the large river systems on Earth^6^, and has changed the characteristics and ecosystem functioning of river systems resulting in severe alterations of riverine carbon processing at the local, regional and global scale^7–9^. River impoundments interrupt the river continuum, and create alternating series of more or less lentic and lotic reaches, as described by the serial discontinuity concept^10^. Impoundments are hotspots for organic carbon sedimentation and mineralization^8,11^, and organic carbon trapping together with high metabolic rates can result in increased greenhouse gas emissions from river impoundments^9,12,13^. However, river impoundments can also be hotspots for CO_2_ uptake by phytoplankton due to high primary production rates^8^, as increased water residence time (WRT), and reduced turbidity favor phytoplankton growth^14,15^. Impoundments alter not only the carbon dynamics upstream of the dam, but also downstream (e.g. by altering organic carbon quality and quantity^16^), and can cause high rates of primary production even downstream of the dam^17^.

Measurements of riverine metabolism (Gross Primary Production and Ecosystem Respiration) can be used to analyze carbon cycling in river networks, as primary production and respiration control large parts of carbon transformation in rivers, but vary in relation to river characteristics and the position along the river continuum^18,19^. Compared to terrestrial and lentic systems, the characterization of flowing waters according to their metabolic regimes is incomplete^20^. Metabolism has been more frequently estimated in streams than in rivers, and comparing the metabolism of flowing waters of different size and characteristics revealed large differences^20^. Moreover, the dynamic nature of flowing waters complicates the comparison of rivers based on their metabolism.

The availability of low-cost dissolved oxygen (DO) sensors, and easy-to-use software for the calculation of gross primary production (GPP) and ecosystem respiration (ER) according to Odum’s^21^ diel oxygen method, enables scientists to estimate riverine metabolism more frequently and in greater detail, which will improve the understanding of metabolic regimes in rivers^20,22^. In order to better understand and describe the variability in riverine metabolism and its effect on carbon processing in river systems, widespread monitoring of rivers of different size, watershed characteristics, and anthropogenic disturbance, such as damming, is needed.

Compared to rivers in cold or tropical regions, phytoplankton biomass in many temperate, anthropogenically influenced river systems is high in relation to the total organic carbon (TOC) load^23,24^. This suggests that phytoplankton CO_2_ uptake plays a significant role in carbon spiraling (the combined processes of cycling and longitudinal transport^25^) along temperate rivers. Comparing inland waters of different WRT but similar total phosphorus concentrations, phytoplankton abundance was found to decrease with decreasing WRT^26^. Thus, the effect of smaller impoundments (i.e. impoundments with shorter WRT) on phytoplankton dynamics may be more subtle than the influences of large reservoirs^27^. Despite their relatively small size, consecutive low-head dams were found to increase heterotrophic carbon processing along four lentic-lotic sections in a mid-size Mediterranean river^28^. At the same time, very little is known about the importance of phytoplankton for carbon processing in rivers with consecutive low-head dams, factors that could strongly influence GPP, and thus phytoplankton CO_2_ uptake.

In this study, we analyzed spatial and temporal patterns in GPP and ER, as well as phytoplankton biomass (chlorophyll *a* concentration; Chl*a*), along a 74.4-km river reach that is intersected by six low-head navigation dams. We assessed variations along the river (horizontal variation), as well as the vertical variation in one of the intermittently stratified impoundments. We hypothesized that GPP and phytoplankton biomass increase along the studied river reach, since the hydro-morphological conditions in the dam headwaters favor phytoplankton growth.

## Materials and Methods

### Study site

**Figure 1:**
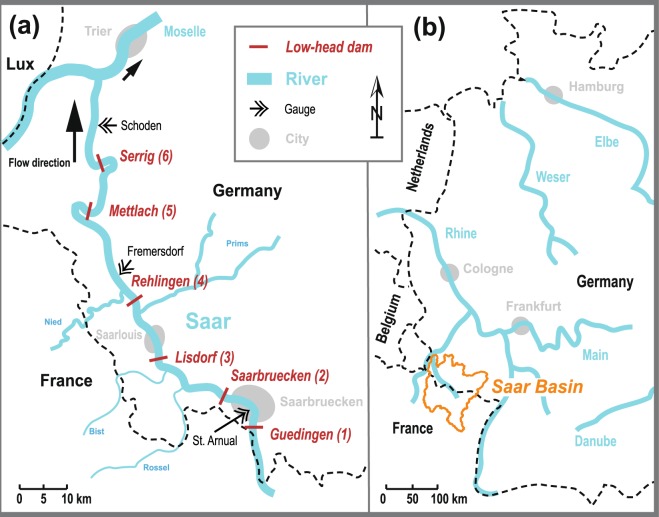
(**a**) German section of the river Saar from the French-German border south of Saarbruecken to its confluence with the river Moselle close to the city of Trier. Shown are the six low-head dams, the main tributaries and cities along the river course. The six low-head dams correspond to the six measuring stations of this study, and are numbered from up- to downstream. (**b**) Location of the Saar basin, which is part of the Rhine basin in western Germany.

The study was carried out in the river Saar, which runs from the Vosges Mountains through France and Germany. After 246 km the river enters the river Moselle which is a tributary of the river Rhine (Fig. 1b). The catchment area of the Saar covers 7452 km^2^, and consists of 49.9% agricultural land, 37.3% forests and semi-natural areas, 12% artificial surfaces (urbanized land), and 0.6% water bodies and wetlands^29^. The long-term annual mean discharge at the representative gauge Fremersdorf (Fig. 1a) is 73.1 m^3^ s^−1^, and the mean annual discharge during the studied years 2014 and 2015 at the gauges St. Arnual, Fremersdorf, and Schoden (Fig. 1a) was 30.4, 55.9 and 56.9 m^3^ s^−1^, respectively^30^. The mean annual discharge of the tributaries Rossel, Bist, Prims, and Nied (Fig. 1a) during 2014 and 2015 was 1.2, 0.6, 8.2, and 8.2 m^3^ s^−1^, respectively, which together amounts to 33% of the discharge in the main stem at Fremersdorf.

Although eutrophication of the Saar has been reduced strongly by improved wastewater treatment during the 1980s, and nutrient loads decreased further until 2002^31^, the degree of pollution in the Saar is still considerable with mean total phosphorus and nitrogen concentrations of 0.16 and 3.1 mg l^−1^, respectively (at the station Guedingen 2014–2015; LUA Saarland, unpublished data). Despite improved wastewater treatment, concomitant declines in phytoplankton biomass between 1990 and 2010 did not occur^31^.

The German section of the river was gradually reconstructed between 1976 and 2000 to improve navigation for cargo shipping purpose. Today, it is regulated by six low-head dams (Fig. 1a) of heights between 2.4 and 14.5 m with installed ship-locks and hydropower plants. The reconstruction included a widening of the river cross section and a deepening of the river channel, and caused a doubling of the average WRT and water depth, resulting in a mean water depth of 4.2 m, and an average WRT of 4.1 days at a discharge of 80 m^3^ s^−1^ for the entire German section of the river^32^. In the largest impoundment (Serrig, Fig. 1a) the river has a maximum width of 170 m, a water depth of up to 11 m, and during low flow in summer flow velocities fall below 0.1 m s^−1^ ^32^. In the deeper impoundments, thermal stratification on a diurnal basis (with a breakdown of stratification during night) occurs during spring and summer. In the impoundment Serrig, a temperature difference of at least 1 °C between the 1 and 4 m water layer occurred at 26% of days during the summer half-year 2014^33^. These dam headwaters are water bodies showing properties of both free-flowing rivers and reservoirs, and are characterized by dynamic changes in mixing conditions.

### Field measurements

We analyzed monitoring data of continuous measurements (48 recordings day^−1^) of chlorophyll *a* concentration (Chl*a*), dissolved oxygen concentration (DO), and water temperature (WT) measured *in situ* with multi-parameter sondes (YSI 6920, YSI inc., USA). The sondes were attached to buoys at water depths between 0.5 and 1 m (Table 1). Deviations in the deployment depth between stations occurred due to slight constructional differences. Chl*a* was measured with a chlorophyll fluorescence sensor (YSI 6025, YSI inc, USA), and DO with an optical DO sensor (YSI 6150, YSI inc., USA). The sensors were calibrated according to the manufacturer’s instructions and cleaned with an automatic wiper.

**Table 1:** Distance of the measuring stations from the confluence with the river Moselle (Location), mean water depth of the cross section at the measuring station at mean discharge (Water depth), depth of sensor deployment (Sensor depth), calculated mean current velocity during the study period 9 April–30 September 2014 and 2015 (Current velocity), mean water temperature during the study period (Water temperature). Above: above the dam, below: below the dam.

Station	Location [river-km]	Water depth [m]	Sensor depth [m]	Current velocity [m s^−1^]	Water temperature [°C]
Guedingen (site 1)	92.7	2.6	0.8	0.29	17.5
Saarbruecken (site 2)	82.2	3.8	0.8	0.11	17.7
Lisdorf (site 3)	66.2	2.8	0.5	0.14	18.2
Rehlingen (site 4)	53.9	3.9	0.6	0.14	18.7
Mettlach (site 5)	31.3	4.3	0.9	0.12	17.2
Serrig (site 6) *above*	18.5	8.2	1.0–4.3	0.06	19.0
Serrig (site 6) *below*	18.3	4.4	0.7	0.10	18.8

For monitoring purposes, six multi-parameter sondes were placed along the river, each downstream of the six dams. They were numbered in accordance with the flow direction (Fig. 1a, and Table 1) the first being at the most upstream and the last at the most downstream location. Data were recorded between 9 April and 30 September 2014 and 2015, i.e., we used measurements from 350 days at each station, at an interval of 30 minutes. Over the entire study period, we had to exclude a total of 30 days of measurements from different stations because of malfunctioning of the sensors (days were excluded when less than 48 recordings day^−1^ were available).

In addition to the data from the six sondes, monitoring data from a seventh multi-parameter sonde, which had been deployed as a profiling system (custom-built model, Germafin Engineering GmbH, Thür, Germany), were used. The profiling system was placed above the dam at Serrig (site 6; Fig. 1a, and Table 1). The sonde was moved vertically by a chain driven mechanism coupled to a metal rail that was suspended in the water. Profiles were collected at two-hourly intervals from the surface, with stops after each 0.5 m, to a water depth of around 4 m, and then back to the surface. The sonde measured at a 4-minute interval, but as depth profiles were recorded at two-hourly intervals, we used 12 measurements per day from each depth for our analysis. For the analysis of vertical differences, measurements from between 244 and 261 days, depending on the depth (days were excluded when less than 12 recordings per depth and day were available), recorded between 9 April and 30 September 2014 and 2015, were used.

Additionally, we used mean daily discharge for the entire study period available from the gauges St. Arnual, Fremersdorf, and Schoden (Fig. 1a). Biweekly data on nutrient concentrations in the Saar and its tributaries were received from the State Agency of Environment of the German federal state of Saarland (LUA Saarland). Briefly, water was sampled in 2014 (n = 24) and 2015 (n = 25) in fully mixed sections of the rivers. Nutrient concentrations (total nitrogen, total phosphorus, ortho-phosphate-P, ammonium-N, and nitrate-N) were determined using German Standard Methods^34^. Data on TOC in the Saar was taken from the monitoring program of the State Ministry of Environment, Energy, Nutrition, and Forestry of the German federal state of Rhineland-Palatinate (MUEEF Rheinland-Pfalz), and can be freely accessed at http://www.geoportal-wasser.rlp.de/servlet/is/2025/.

### Metabolism calculation

We calculated daily GPP and ER (and net ecosystem production, NEP, NEP = GPP−ER) using the diel oxygen method by Odum^21^, which is an estimate on the ecosystem metabolism derived from daily variation in the production and consumption of oxygen, following the equation:

1 $$\frac{{\rm{d}}O}{{\rm{d}}t}={\rm{GPP}}-{\rm{ER}}+K({C}_{s}-C)$$

where $\frac{{\rm{d}}O}{{\rm{d}}t}$ is the rate of change in oxygen concentration, GPP is the gross primary production, ER is the ecosystem respiration, *K* is the reaeration coefficient, *C*_*s*_ is the saturation concentration of oxygen, and *C* is the oxygen concentration at a given time^35^.

For the calculation we used the R software package StreamMetabolism^36^ that computes GPP and ER from diel oxygen curves. StreamMetabolism estimates reaeration from current velocity and water depth using the O’Connor Dobbins surface renewal method^36,37^, following the equation:

2 $${K}=\frac{3.93\,{V}^{0.5}}{DE{P}^{1.5}}$$

where K is the reaeration coefficient, V is the current velocity, and DEP is the water depth. We calculated daily mean current velocity at each station using daily mean discharge, and the cross sectional area at the respective station (Table 1). StreamMetabolism applies a temperature correction for *K* and ER. First, *K*_20_ _°C_ and ER_20_ _°C_ are calculated, and are subsequently corrected for WT.

When using the single station oxygen method, GPP is by definition ≥ 0 and ER ≤ 0, since an inversion of the processes is metabolically impossible^38^. In 20.4% and 15.9% of the analyzed days, we calculated negative GPP and positive ER, respectively (for a detailed description of the causes for calculated negative GPP and positive ER see the discussion section). For the days with negative GPP or positive ER, values for both GPP and ER were excluded from the analysis.

To quantify the carbon processing in the Saar, we calculated the daily rate of carbon dioxide consumption from the daily rate of oxygen production according to:

3 $${\rm{g}}\,{\rm{C}}={\rm{g}}\,{{\rm{O}}}_{2}\times \frac{1}{PQ}\times \frac{12}{32}$$

where PQ is the photosynthetic quotient (mol O_2_ released during photosynthesis/mol CO_2_ incorporated), 12 is the atomic mass of carbon, and 32 is the molecular mass of oxygen^39^. Further, we calculated the daily rate of carbon dioxide production from the daily rate of oxygen consumption according to:

4 $${\rm{g}}\,{\rm{C}}={\rm{g}}\,{{\rm{O}}}_{2}\times RQ\times \frac{12}{32}$$

where RQ is the respiratory quotient (mol CO_2_ released/mol O_2_ consumed), 12 is the atomic mass of carbon, and 32 is the molecular mass of oxygen^39^. We used a PQ of 1.25 as phytoplankton in the Saar takes up both ammonium and nitrate^33,40^, and a PQ of 1.25 was found to be applicable for a river in the same geographical region^41^. We further used a RQ of 1 as it lies between the suggested RQ of 1.2 for lakes^42^, and of 0.85 for streams^39^.

Additionally, we calculated the photosynthetic capacity (GPP:Chl*a* ratio) of phytoplankton for all stations along the studied reach by dividing the oxygen production by phytoplankton by Chl*a*.

According to Odum^21^ the single station oxygen method requires the assumption of stream homogeneity upstream of the zone of measurement, when the metabolism of one particular point along the stream is to be measured. In the case of stream homogeneity upstream, it can be assumed that the water passing the measuring station throughout the day has had the same diurnal history along its entire flow path^21^. In natural as well as anthropogenic influenced rivers this assumption is rarely fulfilled. Over the course of a day water parcels are flowing through river reaches of different characteristics, and thus the metabolism calculated with the single station method reflects the heterogeneous upstream conditions^43^. However, this does not imply that the diel oxygen method cannot be applied under heterogeneous upstream conditions, like in our study, with sites below dams. It rather means that the calculated metabolism reflects the cumulative effects of the upstream conditions. Thus, our calculated metabolism does not solely reflect the metabolism at the point of measurement, but includes the combination of conditions at the dam and its headwater^18,43^. Consequently, measuring below the dams allowed us to integrate the effect of the impoundments in our analysis of the river’s metabolism. However, it has to be noted that during high flow the effects of several dams and the respective headwater reaches might have overlapped. As absolute GPP and site-to-site variation during high flow was low, GPP during high flow periods had a minor influence on the overall results.

Another precondition for the use of the Odum’s single station method in streams and rivers is vertical homogeneity within the water column. As the slow-flowing sections of the deep impoundments at the Saar have been found to periodically stratify during spring and summer (see section on study site), the assumption of vertical homogeneity was during some days in the deeper impoundments temporarily breached (e.g. in the impoundment Serrig, a temperature difference of at least 1 °C between the 1 and 4 m water layer was reported during 26% of days during the summer half-year 2014^33^). To assess the degree of possible bias due to reduced oxygen supply to the water column, we performed a sensitivity analysis, in which we re-ran the metabolism calculation while setting reaeration to zero. This allowed us to quantify the maximum possible error in the metabolism calculations for the case zero oxygen supply to the water column.

### Statistical analyses

To test for differences in GPP between the measurement stations, and for differences in GPP between different months we applied a Friedman Rank Test^44^. The Friedman Rank Test is a non-parametric test for finding differences between multiple groups. It ranks the data in each group creating a table of ranks, and then computes the test statistic from the mean ranks of the groups^44^. We used a non-parametric test since normal distribution, tested with a Shapiro-Wilk test for normality, was not given, and could not be achieved by transformation. A test for paired groups (Friedman Rank Test) was used, since the compared groups were not independent of each other. To test for correlation between mean daily GPP and ER we used the non-parametric Kendall’s tau coefficient. To test for a relation between mean daily GPP and vertical differences in WT in the headwater of the dam Serrig (site 6), we calculated the daily mean WT difference between the 1 and 4 m water layer, and related this difference to the mean daily GPP using Kendall’s tau coefficient.

The calculation of ecosystem metabolism from diel oxygen curves, and the Friedman Rank Test were carried out using the software R version 3.4.2 (R Core Team, Vienna, Austria). All other analyses were performed using JMP, version 12.0.1 (SAS Institute Inc., Cary, NC, USA).

## Results

### Variability in GPP and phytoplankton biomass along the Saar

**Figure 2:**
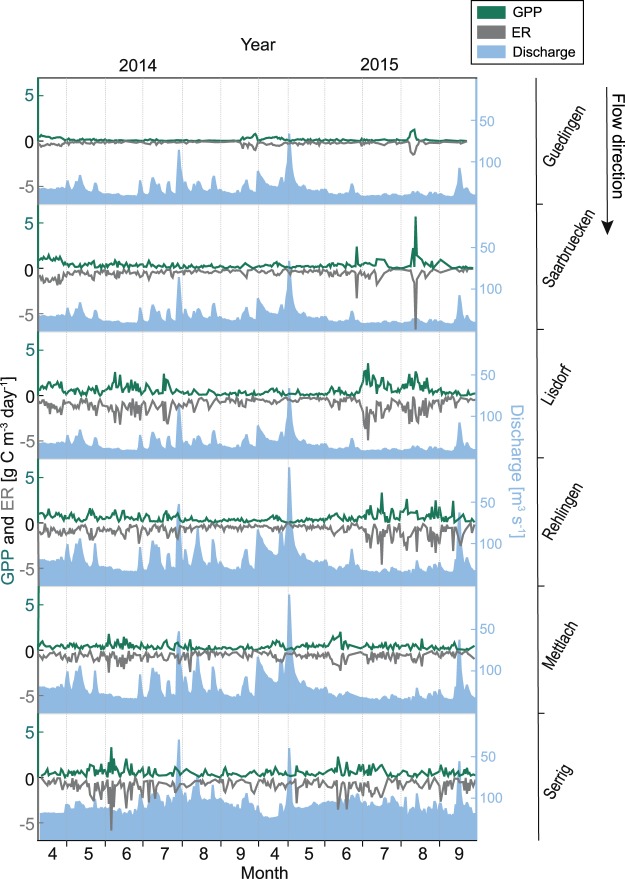
Time-series of gross primary production (GPP), ecosystem respiration (ER), and daily mean discharge for the six measuring stations along the Saar. Values for the period 9 April–30 September are shown for the years 2014 and 2015. For the stations Guedingen, Saarbruecken, and Lisdorf discharge from the gauge St. Arnual, for the stations Rehlingen and Mettlach discharge from the gauge Femersdorf, and for the station Serrig discharge from the gauge Schoden are displayed (cf. Figure 1a).

Mean GPP along the studied river reach increased from 0.18 g C m^−3^ d^−1^ at the upstream site Guedingen (site 1) to 0.63 g C m^−3^ d^−1^ at the station Lisdorf (site 3), and decreased slightly downstream (Table 2 and Fig. 2). At the downstream end of the study reach at Serrig (site 6) mean GPP was 0.56 g C m^−3^ d^−1^. The difference between Guedingen (site 1) and Lisdorf (site 3) constitutes an increase of 0.45 g C m^−3^ d^−1^ over a distance of 26.5 km. Taking the calculated GPP during the study period from all sites into consideration a significant difference in GPP between sites was observed (Friedman Rank Test, P < 0.0001), mainly due to substantially different GPP at Guedingen (site 1) and Saarbruecken (site 2) (Table 2).

**Table 2:** Calculated gross primary production (GPP) and ecosystem respiration (ER) for the six stations along the river Saar (9 April–30 September 2014 and 2015). Since an inversion of the processes is metabolically impossible, we excluded days with calculated negative GPP and positive ER from the analysis (GPP and ER in columns 3 and 4). In column 6 and 7, days with calculated negative GPP and positive ER were set to zero and included in the average value (see discussion for further details). Numbers in brackets indicate the standard deviation.

Station	Number of days	Mean GPP [g C m^−3^ d^−1^]	Mean ER [g C m^−3^ d^−1^]	Number of days	Mean GPP (days with negative values set to zero) [g C m^−3^ d^−1^]	Mean ER (days with positive values set to zero) [g C m^−3^ d^−1^]
Guedingen (site 1)	217	0.18 (±0.19)	−0.26 (±0.22)	344	0.12 (±0.18)	−0.17 (±0.21)
Saarbruecken (site 2)	266	0.38 (±0.47)	−0.55 (±0.55)	350	0.29 (±0.44)	−0.43 (±0.53)
Lisdorf (site 3)	294	0.63 (±0.59)	−0.99 (±0.73)	349	0.53 (±0.59)	−0.86 (±0.74)
Rehlingen (site 4)	304	0.57 (±0.46)	−0.80 (±0.62)	347	0.50 (±0.46)	−0.71 (±0.63)
Mettlach (site 5)	272	0.42 (±0.32)	−0.65 (±0.43)	335	0.35 (±0.32)	−0.54 (±0.45)
Serrig (site 6)	229	0.56 (±0.46)	−0.90 (±0.77)	345	0.38 (±0.45)	−0.61 (±0.75)
Total Mean	1582	0.46 (±0.46)	−0.71 (±0.63)	2070	0.36 (±0.50)	−0.55 (±0.62)

Mean Chl*a* increased from 4.6 µg l^−1^ at Guedingen (site 1), with a decline between Lisdorf (site 3) and Rehlingen (site 4), to 13.3 µg l^−1^ at Serrig (site 6), which constitutes an increase of 0.12 µg l^−1^ river-km^−1^ (Fig. S1a). In contrast to GPP, highest mean Chl*a* was found in the downstream part of the study reach at Serrig (site 6) (Table 2 and Fig. S1a). The variation of GPP and Chl*a* along the river differed slightly. The magnitude of both mean GPP and Chl*a* was lowest at Guedingen (site 1) and increased downstream. While GPP reached highest mean values at the station Lisdorf (site 3), and decreased slightly downstream, Chl*a* was highest at the downstream stations Mettlach (site 5) and Serrig (site 6) (Table 2 and Fig. S1a). Thus, the mean oxygen production by phytoplankton per unit biomass (i.e. the quotient GPP:Chl*a*) increased from 5 mg O_2_ mg^−1^ Chl*a* h^−1^ at Guedingen (site 1) to 34 mg O_2_ mg^−1^ Chl*a* h^−1^ at Rehlingen (site 4), and distinctly dropped downstream of Rehlingen (site 4) showing values of ~10 mg O_2_ mg^−1^ Chl*a* h^−1^.

**Figure 3:**
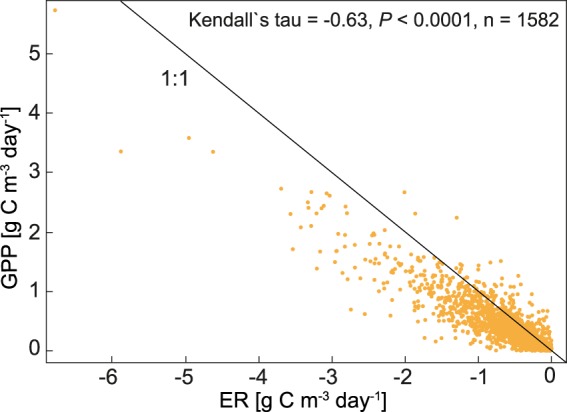
Correlation between the mean daily gross primary production (GPP), and the mean daily ecosystem respiration (ER) from all stations along the Saar. Data from the period 9 April–30 September 2014 and 2015.

Variation in the two-year mean ER along the river showed the same pattern as variation in the two-year mean GPP with lowest rates upstream (−0.26 g C m^−3^ d^−1^ at Guedingen, site 1; Table 2), and highest rates in the middle part of the study reach (−0.99 g C m^−3^ d^−1^ at Lisdorf, site 3; Table 2). On a daily basis, GPP and ER were correlated (Fig. 3). During the study period mean ER exceeded mean GPP at all stations, i.e. the studied river reach was a net heterotrophic system (Table 2). At all stations, days at which GPP exceeded ER, i.e. during these days the river was net autotrophic, occurred (between 10 and 18% of days).

### Vertical variability in GPP and phytoplankton biomass in the impoundment Serrig

**Figure 4:**
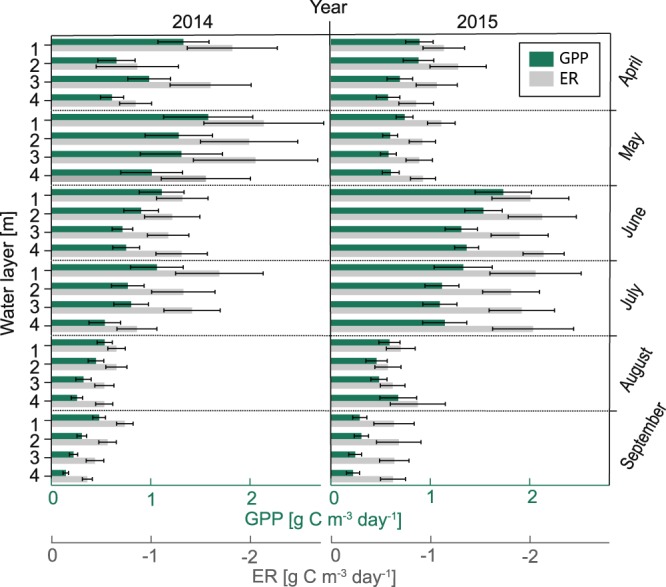
Monthly mean gross primary production (GPP) and ecosystem respiration (ER) for the years 2014 and 2015 in the one, two, three and four meter water layer above the dam Serrig (site 6). Error bars show the standard error of the respective monthly mean GPP and ER.

In the impoundment Serrig (site 6), vertical variation in GPP and ER were present during all months (Fig. 4). Analyzing GPP and ER in the upper 4 m of the water column over the entire study period, we measured greatest values for mean GPP and ER of 1.04 and −1.45 g C m^−3^ d^−1^, respectively (median: 0.75 and −0.99 g C m^−3^ d^−1^, respectively), in the 1 m water layer, while lowest mean values for GPP and ER of 0.73 and −1.19 g C m^−3^ d^−1^, respectively (median: 0.51 and −0.89 g C m^−3^ d^−1^, respectively), occurred in the 4 m water layer. Hence, GPP between the 1 and 4 m water layer differed on average by a factor of 1.4 (median: 1.5), and showed an absolute difference of 0.31 g C m^−3^ d^−1^ (median: 0.23 g C m^−3^ d^−1^). Mean GPP and ER over the entire upper 4 m of the water column amounted to 0.86 and −1.31 g C m^−3^ d^−1^, respectively (median: 0.60 and −0.93 g C m^−3^ d^−1^, respectively). Comparing different months and years, mean monthly GPP was mostly highest in the 1 m water layer, and decreased for most months gradually from 1 to 4 m water depth (Fig. 4). Days with positive net ecosystem production (NEP) occurred at all four depths and was not restricted to certain months. However, the number of days with positive NEP decreased from 22% in the 1 m, to 11% in the 4 m water layer.

**Figure 5:**
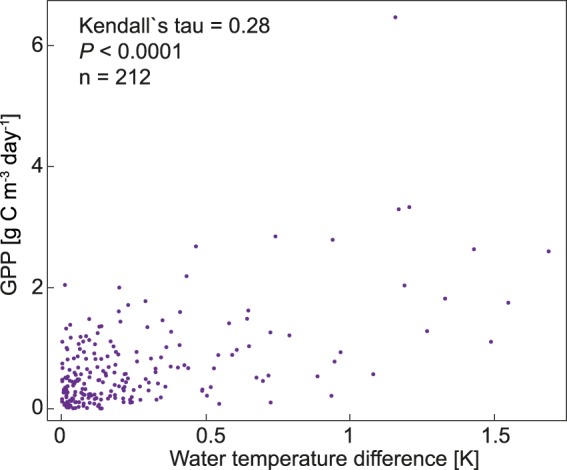
Correlation between the daily mean water temperature difference between the one and the four meter water layer and the daily mean gross primary production (GPP) over the entire upper 4 m of the water column in the headwater of the dam Serrig. Data from the period 9 April–30 September 2014 and 2015.

Mean Chl*a* between the 1 and 4 m water layer differed by a factor of 1.2 (median: 1.1), with a mean Chl*a* of 11.7 and 10.1 µg l^−1^ at the 1 and 4 m water layer, respectively (median: 6.8 and 6.2 µg l^−1^, respectively). Thus, the mean GPP:Chl*a* ratio decreased with depth, from 14.2 mg O_2_ mg^−1^ Chl*a* h^−1^ in the 1 m to 13.5 mg O_2_ mg^−1^ Chl*a* h^−1^ in the 4 m water layer (median: 11.4 and 8.1 mg O_2_ mg^−1^ Chl*a* h^−1^, respectively). Mean daily GPP over the entire upper 4 m of the water column was positively correlated with the mean daily difference in WT between the 1 and 4 m water layer (Fig. 5).

### Temporal variability in GPP and phytoplankton biomass

**Figure 6:**
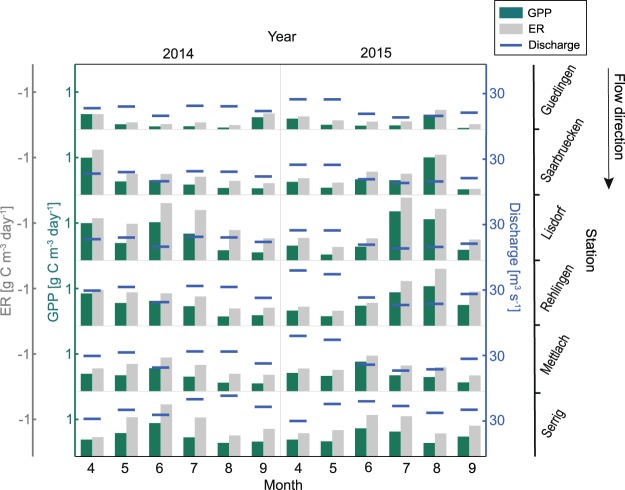
Spatio-temporal distribution of mean monthly gross primary production (GPP), ecosystem respiration (ER), and discharge along the river Saar. Data from the period 9 April–30 September 2014 and 2015. For the stations Guedingen, Saarbruecken, and Lisdorf discharge values from the gauge St. Arnual, for the stations Rehlingen and Mettlach discharge values from the gauge Fremersdorf, and for the station Serrig discharge values from the gauge Schoden were used (cf. Fig. 1a).

The calculated GPP over all stations differed significantly between months (Friedman Rank Test, *P* < 0.0001). The intra-annual development of GPP varied strongly between different stations and years (Fig. 6), with differences in monthly mean GPP between years for the same month and station of between 0.001 and 0.83 g C m^−3^ d^−1^. A coherent seasonal pattern of GPP and ER was not apparent when comparing mean monthly GPP and ER between stations and years (Fig. 6). While for some stations in 2014, GPP was highest in spring, highest GPP for the same stations occurred in 2015 during late summer (Fig. 6). For 29% of the days in April the river was net autotrophic (i.e. GPP exceeded ER), while the days with net autotrophy during the other months ranged between 9 and 15%.

**Figure 7:**
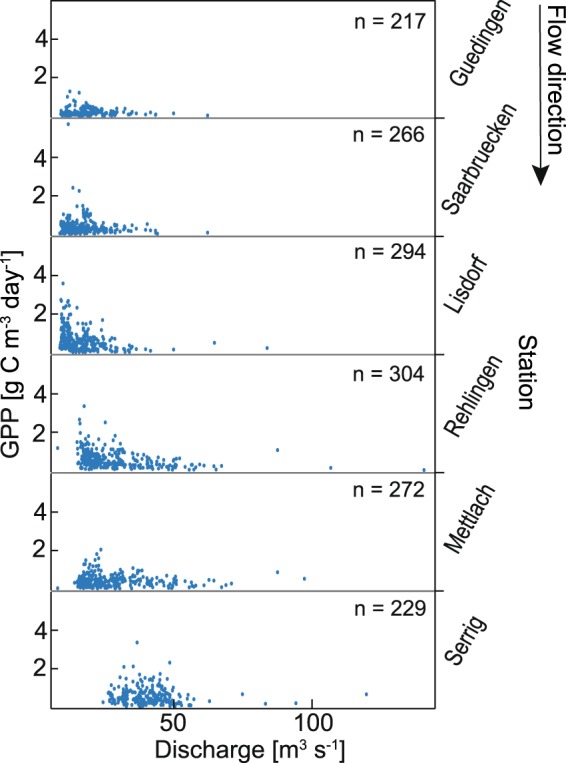
Relationship between daily mean gross primary production (GPP), and daily mean discharge for the measuring stations along the river Saar (9 April–30 September 2014 and 2015). For the stations Guedingen, Saarbruecken, and Lisdorf discharge values from the gauge St. Arnual, for the stations Rehlingen and Mettlach discharge values from the gauge Fremersdorf, and for the station Serrig discharge values from the gauge Schoden were used (cf. Fig. 1a).

High GPP occurred at days with low mean daily discharge (Fig. 7), but days with low GPP even occurred during days with low discharge (Fig. 7). Comparing daily GPP and daily mean WT for single months, GPP was positively (but weakly) correlated with WT (Kendall’s tau for the respective months between 0.13 and 0.33, *P* < 0.001).

### Sensitivity of the metabolism calculation to reaeration estimates

The performed sensitivity analysis, in which we re-ran the metabolism calculation while setting reaeration to zero (see Methods), revealed that the mean GPP per station calculated with zero reaeration differed from our routine calculations by between 2 and 11%, with a greater deviation at the station with higher current velocities (Guedingen, site 1). For ER the mean deviation from the routine calculation per station was between 21 and 37%. For the analysis of vertical differences in the headwater of the dam Serrig (site 6), the sensitivity analysis revealed a mean difference between calculations with and without reaeration of 39 and 56% for mean GPP and ER, respectively. The median deviation between the calculations was merely 2% and 12% for GPP and ER, respectively.

## Discussion

### Factors controlling GPP and phytoplankton biomass in the Saar

The magnitude of GPP increased by 3.5-fold between the stations Guedingen (site 1) and Lisdorf (site 3), resembling GPP of low-production rivers at the upstream site, and of medium-production rivers in the middle and downstream part of the study reach. The strong increase in GPP within several tens of kilometers is surprising as it spans a wide range of the reported variability in riverine GPP^45^. We suggest that the increase in GPP and Chl*a* along the Saar is likely a consequence of the cumulative effect of the consecutive impoundments that increase WRT, light availability and WT, and thus stimulate GPP. Increased GPP in impounded river reaches is a consequence of the disruption of the river continuum (serial discontinuity concept^10^) that results in increased carbon processing, and at the same time slower carbon transport along the river, suggesting a shortened carbon spiraling length.

A similarly strong increase in GPP was found along a 37-km river reach of a small Appalachian river (Little Tennessee River, mean discharge at the upstream site 7.4 m^3^ s^−1^) that strongly increased in size along the studied reach (change from 4th- to 6th-order)^46^. An increase in GPP from small to medium sized rivers has been described by the river continuum concept^19^. In contrast, in a small to medium sized, unshaded New Zealand grassland river (Taieri River, downstream discharge of 37 m^3^ s^−1^), GPP was found to decrease along the river continuum^47^. We suggest that the GPP increase along the Saar is a consequence of the changed river morphology and increased WRT, caused by the cascading impoundments, rather than a result of changes in river size.

GPP along the Saar did not increase steadily, but increased strongly between Guedingen (site 1) and Lisdorf (site 3), and subsequently decreased slightly (Table 2, Fig. 2). The increase in GPP between Guedingen (site 1) and Lisdorf (site 3) was likely a consequence of the prolonged WRT. In the shallower upstream impoundments light availability might be sufficient for high GPP, while in the deeper impoundments phytoplankton might be light limited, so that GPP did not increase further between Lisdorf (site 3) and Serrig (site 6), while phytoplankton biomass remained high. The relationships between the limiting factors light and WRT did likely not only change along the river, but also in the course of the year.

The tributaries Rossel, Bist, Prims, and Nied (Fig. 1a) added considerable nutrient loads to the Saar (mean total nitrogen and phosphorus concentration in the Saar at Guedingen, and of Rossel, Bist, Prims, and Nied was 3.1 and 0.16, 4.7 and 0.33, 3.1 and 0.17, 3.2 and 0.16, 4.0 and 0.15 mg l^−1^, respectively, resulting in a total nitrogen and phosphorus load of 2962 and 159, 183 and 13, 58 and 3, 832 and 41, 1035 and 40 tons year^−1^, respectively, during 2014 and 2015, LUA Saarland, unpublished data). The nutrient loads from the tributaries did probably not stimulate phytoplankton growth in the nutrient-rich Saar. As levels of bioavailable nutrients in the Saar are high even during periods with high phytoplankton biomass (e.g. 0.078, 0.014, and 2.12 mg l^−1^, ortho-phosphate-P, ammonium-N, and nitrate-N, respectively, during a phytoplankton bloom in June 2015 in the impoundment Serrig, site 6)^31,33^, an additional stimulation by nutrient inputs from the tributaries is not likely. Phytoplankton grazing by benthic filter feeders and zooplankton has previously been shown to be low in the Saar compared to other rivers, and thus had likely a minor influence on phytoplankton biomass^48,49^.

Thermal density stratification during spring and summer influenced the spatio-temporal variation in GPP, since we found mean daily GPP in the headwater of the dam Serrig (site 6) to be positively correlated with the magnitude of the mean daily vertical WT difference (Fig. 5). Thus, we suggest that high rates of GPP in the Saar are also a consequence of the stratification events in the dam headwaters. Using the headwater of the dam Serrig (site 6) as an example, we showed that mean GPP was higher in the upper water layers (Fig. 4), indicating that stratification events resulted in vertical differences in mean GPP in the deep impoundments. The vertical gradient in mean GPP underlines the partly reservoir-like characteristics of the slowly flowing river sections. High GPP in the intermittently stratified impoundments was likely a result of improved light conditions for phytoplankton during stratification, and reduced grazing pressure by benthic filter feeders, as the upper water layers were during stratification disconnected from the benthic zone^14,33,48^.

### The metabolic regime of the Saar

The metabolic regime of a river has previously been defined as its characteristic temporal pattern of GPP and ER^20^. We found the metabolism along the impounded Saar to be highly variable, both on the temporal (inter- and intra-annually) and spatial (variation between the measuring stations) scale (Figs 2 and 6), with significant differences in mean GPP between most stations and months, respectively. The spatial and temporal variability in GPP and ER in the Saar make it difficult to identify a coherent metabolic regime for the entire study reach or single stations. The high temporal variation in GPP in the Saar was probably partly a consequence of the variability in discharge (e.g. Young and Huryn^47^; Fig. 7), since low discharge rates reduce turbulence induced turbidity, and may also allow for intermittent vertical stratification that will improve light conditions for phytoplankton, allowing the development of higher GPP and phytoplankton biomass.

We found the daily mean GPP and ER to be correlated (Fig. 3), which is common for river systems^20,45^. This indicates that a large fraction of the mineralized organic matter in the Saar might be of autochthonous origin. This interpretation is supported by the high mean GPP:ER ratio of 0.79 ± 1.26 (error is the standard deviation) in our study that exceeds the average GPP:ER ratio of 0.42 ± 0.14 reported from 37 river sites distributed over different biomes^45^, and is higher than the average GPP:ER ratio in reservoirs^8^. Even if this comparison has to be interpreted with some caution, since we analyzed data from April to September while Finlay^45^ (GPP in rivers), and Maavara *et al*.^8^ (primary production in global reservoirs) report annual GPP and ER, we still consider the GPP:ER ratio in our study as high for rivers. Further, the TOC concentration in the Saar is moderate (mean annual TOC concentration at Serrig during 2015 was 4.8 mg C l^−1^; MUEEF Rheinland-Pfalz, unpublished data), which together with the high GPP:ER ratio suggests that phytoplankton CO_2_ uptake is an important flux for carbon processing in the Saar.

As the phytoplankton CO_2_ uptake per unit biomass varies depending on the environmental conditions, we assessed the GPP:Chl*a* ratio along the river in order to draw conclusions on the photosynthetic capacity of phytoplankton. We found an increase in the mean GPP:Chl*a* ratio along the river between Guedingen (site 1) and Rehlingen (site 4) that might have been caused by a number of processes ranging from adaptation to the local conditions (i.e. determined by light availability, and WRT), to changes in the phytoplankton community. The observed values for the GPP:Chl*a* ratio of between 5 and 34 mg O_2_ mg^−1^ Chl*a* h^−1^ lie roughly within the range of photosynthetic capacities reported for natural phytoplankton populations of 0.5 to 30 mg O_2_ mg^−1^ Chl*a* h^−1^ ^50^. The relatively large range in the GPP:Chl*a* ratio observed along the river indicates that water movement was slow enough to allow for phytoplankton adaptation, i.e. the CO_2_ uptake per unit biomass changed along the river due to changes in environmental conditions.

### Uncertainties in the metabolism estimates

In our analysis, we assumed that the metabolism calculations were not influenced by inflowing groundwater, since the water budget is closed for the discharges measured upstream at the gauge St. Arnual, in the tributaries, and at the downstream gauge at Schoden (German Federal Institute of Hydrology – BfG, unpublished data). Further, we suggest that the riverine GPP mainly originates from phytoplankton, since the river is mostly deeper than the euphotic depth^33^, and thus we presumed benthic primary production to be minor. Likewise, channelization and bank stabilization of the river allow the assumption that primary production by littoral plants, in comparison to pelagic primary production, is minor. Consequently, we believe that the calculation of GPP with the diel oxygen method provided reasonable estimates of phytoplankton CO_2_ uptake in the Saar.

When calculating metabolism from diel DO curves, GPP is by definition ≥ 0 and ER ≤ 0, as an inversion of the processes is metabolically impossible^38^. In 20.4% and 15.9% of the analyzed days, we calculated negative GPP and positive ER, respectively. A greater number of days with negative GPP and positive ER occurred at stations where the mean GPP and ER over the entire study period were comparatively low (35% of days with GPP < 0 at Guedingen, site 1, and 27% of days with ER > 0 at Serrig, site 6). Accordingly, fewer days with negative GPP and positive ER occurred at stations where the total mean GPP and ER over the entire study period was high (8.6% of days with GPP < 0 at Rehlingen, site 4, and 5.4% of days with ER > 0 at Lisdorf, site 3).

Calculated negative GPP and positive ER were a consequence of weakly defined DO curves (low differences in DO between night and day, increases in DO during night, or decreases in DO during day). Low DO differences between day and night might be a consequence of low metabolic activity during high flow. DO increases during night in the tailwater of the dams were likely a consequence of mixing of the water column in the dam headwaters during night, in cases where thermal stratification occurred at daytime. During stratification DO concentrations in the deeper water layers of the dam headwaters are often low^32^, and mixing with DO enriched surface water during night can result in increasing DO concentrations in the dam tailwater during nights that follow days with stratification.

Previously, it has been assumed that calculated negative values for GPP and positive values for ER are part of measurement imprecision, and when including these values in average estimates under- and overestimates would cancel each other out^51^. Recently, it has been shown that the inclusion of days with negative GPP when calculating long-term mean GPP is not cancelled out by overestimation of GPP during other days, and thus phytoplanktonic GPP is underestimated^38^. Therefore, we excluded the days with GPP < 0 and ER > 0 from our analysis in accordance to earlier studies on streams and rivers^23,52^. Another reasonable way of dealing with negative GPP and positive ER values is to set GPP and ER for these days to zero. The underlying assumption of this procedure is that calculated negative GPP and positive ER occur at days with low GPP and ER, because of weakly defined DO curves, and setting GPP and ER to zero for these days would result in a lower bias of the long-term average GPP and ER than excluding these days from the analysis. As the standard procedure is to exclude days with negative GPP and positive ER from the analysis, we followed this procedure but show average values for both procedures, excluding days with negative GPP and positive ER, and setting the values at these days to zero (Table 2). Neither the in- or exclusion of days with negative GPP or positive ER, nor setting GPP or ER at these days to zero had an influence on the patterns and conclusions described in this study.

At low discharge, the metabolism calculation might have been biased, since at low discharge oxygen supply to deeper water layers might have periodically been reduced, because of intermittently occurring stratification events in the deep impoundments. In the analysis on the sensitivity of the metabolism calculations to reaeration, we found the mean GPP per station calculated with zero reaeration to differ from the routine calculation by between 2 and 11%. A high deviation occurred at the station with higher flow velocities (Guedingen, site 1), for which vertical homogeneity can be assumed. Consequently, miscalculation of mean GPP due to reduced oxygen supply to deeper water layers was 6% at the most, and we concluded that it did not cause strong bias. These findings coincide with an earlier analysis on the influence of the reaeration coefficient on uncertainty in metabolism calculations, which showed that uncertainty in metabolism estimates was low when the reaeration coefficient was low^53^ (i.e. at low current velocity and greater water depth). The reaeration coefficient for the stations along the Saar was low, ranging between 0.06 and 0.85 d^−1^.

For the analysis on vertical variability in GPP and ER the calculation with zero reaeration produced a larger bias (39 and 56% for mean GPP and ER, respectively). To reduce the error in the provided values we reported both mean and median values for the analysis on vertical variations (see results), since median values are less biased by extreme values.

### Significance of phytoplankton CO_2_ uptake for carbon processing in impounded river systems

For oceans and lakes it is well established that phytoplankton CO_2_ uptake can influence the partial pressure of CO_2_ (*p*CO_2_) in water, and thus CO_2_ emissions^54,55^. The simultaneous increase in GPP and ER along the Saar (GPP and ER increased between Guedingen, site 1, and Lisdorf, site 3 by a factor of 3.5 and 3.8, respectively) suggests that cascading impoundments might stimulate both CO_2_ uptake by phytoplankton, and CO_2_ production by mineralization of organic matter, so that the respective increases might balance each other out. Previously, the impoundments along the Saar have been found to be methane emission hot spots^12^. This suggests that a fraction of the organic matter that is not mineralized in the water column or transported downstream leaves the system after burial and mineralization in the sediments as methane. Nevertheless, the mean GPP:ER ratio in the Saar is high compared to global rivers^45^ and reservoirs^8^, illustrating the importance of phytoplankton CO_2_ uptake for the carbon dynamics in the Saar.

Along the entire river reach studied (comparison between Guedingen, site 1, and Serrig, site 6) GPP more than doubled. However, it has to be kept in mind that the effect of dams on riverine phytoplankton dynamics varies strongly between rivers. Phytoplankton gross primary production and biomass in impounded river reaches might even decrease when the water column in the impoundment does not stratify, and impoundments are too dark and deep for substantial phytoplankton growth. In contrast, even in undammed rivers phytoplankton biomass can strongly increase along the river course^56^.

Assuming a mean euphotic depth of 3 m^33^, and GPP during winter to be low in the Saar, we calculated a mean phytoplankton CO_2_ uptake over all stations (based on the measurements from the period April to September shown in Table 2) of 242 g C m^−2^ yr^−1^. This number is in the range of the average CO_2_ uptake by phytoplankton GPP in global lakes of 260 g C m^−2^ yr^−1^ ^57,58^, and slightly higher than the mean organic carbon burial in global reservoirs of 169 g C m^−2^ yr^−1^ ^11^, the mean CO_2_ emission from global hydroelectric reservoirs of 150 g C m^−2^ yr^−1^ ^9^, and from global lakes and reservoirs of 106 g C m^−2^ yr^−1^ ^59^. Further, it exceeds for example the average carbon consumption by phytoplankton primary production in the tropical Congo river of about 62 g C m^−2^ yr^−1^ ^60^, but is clearly lower than carbon consumption by primary production in desert streams (up to 5400 g C m^−2^ yr^−1^ ^61^), or the mean CO_2_ emissions from the global river network of approximately 849 g C m^−2^ yr^−1^ ^5^, and mean CO_2_ emission from Swedish low order streams which was found to amount to ~3800 g C m^−2^ yr^−1^ ^62^.

Overall, our study contributes to a better understanding of aquatic metabolism and CO_2_ dynamics in anthropogenically impacted river systems. We showed that phytoplankton CO_2_ uptake is an important, but highly variable carbon flux in the Saar, which highlights the importance of phytoplankton for carbon processing in impounded river systems. Our study also demonstrates the value of high-frequency water quality monitoring, since lower frequency measurements would not allow calculations of representative daily, seasonal and inter-annual metabolism rates and carbon fluxes. For a better understanding of carbon processing during transport from land to sea along oftentimes intermittent river continua, the incorporation of phytoplankton CO_2_ uptake into riverine carbon budgets is crucial.

## Supplementary information

Supplementary Material

## Data Availability

Correspondence and requests for materials should be addressed to Fabian.Engel@ebc.uu.se.
