# Supplementary material for: Phytoplankton gross primary production increases along cascading impoundments in a temperate, low-discharge river: Insights from high frequency water quality monitoring
Source: Sci Rep. 2019 Apr 30;9:6701. doi: 10.1038/s41598-019-43008-w (PMC6491547; doi:10.1038/s41598-019-43008-w)
Supplement: Supplementary file 1 — Supplementary Material [file 41598_2019_43008_MOESM1_ESM.docx]

Supplementary Material

Phytoplankton gross primary production increases along cascading impoundments in a temperate, low-discharge river: Insights from high frequency water quality monitoring

Fabian Engel^1*^, Katrin Attermeyer^1,2^, Ana I. Ayala^1^, Helmut Fischer^3^, Volker Kirchesch^3^, Don C. Pierson^1^, Gesa A. Weyhenmeyer^1^

^1^*Department of Ecology and Genetics/Limnology, Uppsala University, Norbyvägen 18D, 752 36 Uppsala,* *Sweden.*

^2^*current address: WasserCluster Lunz Biologische Station GmbH, Dr. Carl Kupelwieser Promenade 5, 3293 Lunz am See, Austria*

^3^*Department of Microbial Ecology, German Federal Institute of Hydrology (BfG), Am Mainzer Tor 1, 56068 Koblenz, Germany*

**e-mail: Fabian.Engel@ebc.uu.se*


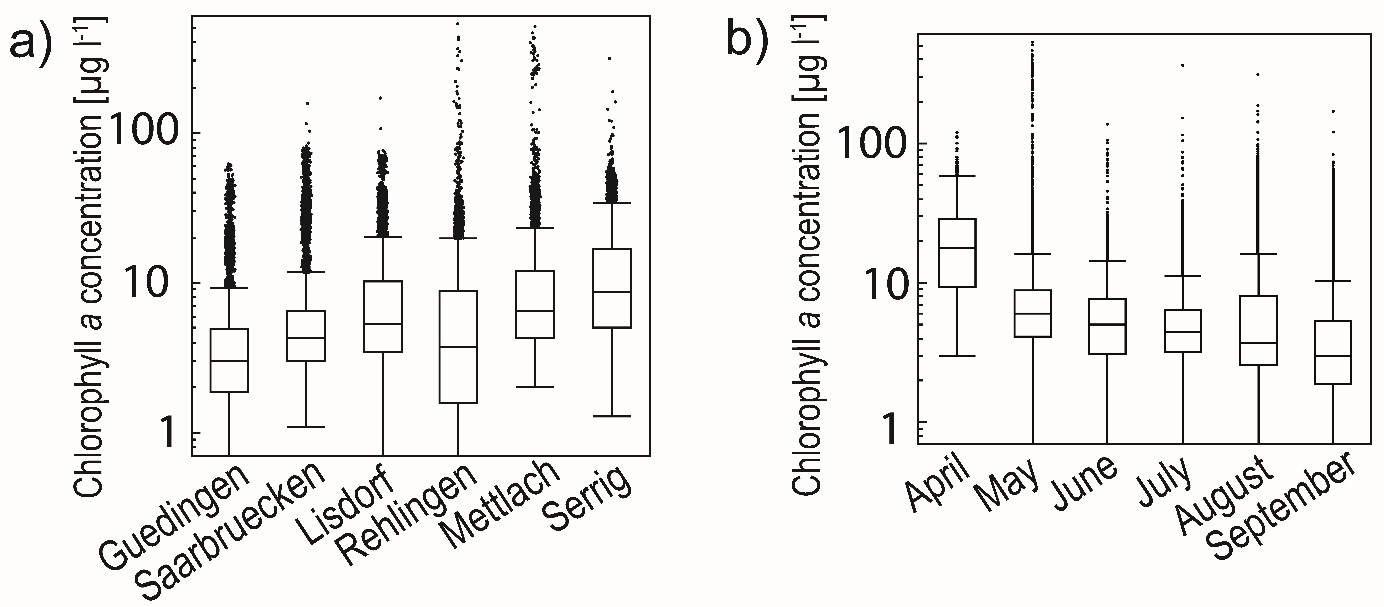


Figure S1. Spatio-temporal distribution of the chlorophyll a concentration (Chla) in the river Saar (data from the period 9 April – 30 September 2014 and 2015). a) Chla at the different measuring stations, b) Chla for different months from all stations. The central line represents the median, the ends of the boxes represent the first and the third quartile, and the whiskers extend from the ends of the boxes to the outermost data point that falls within 1.5-times the inter quartile range. Both y-axes are displayed on a logarithmic scale. The lower whisker of some boxplots are lying outside of the diagram area as they were approaching zero, and thus could not be displayed on a logarithmic scale.
